# Perioperative Management of Parturient Women With Severe Pulmonary Arterial Hypertension Secondary to Atrial Septal Defect

**DOI:** 10.7759/cureus.48369

**Published:** 2023-11-06

**Authors:** Mohammad Nizam Mokhtar, Raha Abdul Rahman, Nadia Md Nor, Azarinah Izaham, Syarifah Noor Nazihah Sayed Masri

**Affiliations:** 1 Department of Anaesthesiology & Intensive Care, Faculty of Medicine, Universiti Kebangsaan Malaysia, Kuala Lumpur, MYS

**Keywords:** atrial septal defect, lower segment caesarean section, anesthesia, pulmonary artery hypertension, pregnancy

## Abstract

At our institution, we occasionally see pregnant patients in the later stages of pregnancy who present with severe pulmonary arterial hypertension caused by congenital heart disease. The physiological changes in pregnancy may worsen the cardiovascular status leading to heart failure which is associated with a high incidence of morbidity and mortality. A scheduled caesarean section in such patients ensures delivery in a controlled environment, avoiding prolonged labour, which is detrimental. Perioperative complications that may worsen pulmonary arterial hypertension should be prevented. The perioperative management, in particular, the anaesthesia technique used and the clinical outcome of this population, is discussed through five interesting cases. Despite a multidisciplinary team and intensive care management, two patients with existing cor pulmonale, one of whom received general and the other central neural blockade anaesthesia, succumbed to their illness immediately after lower segment caesarean section. The management of severe pulmonary arterial hypertension in pregnant patients remains a multidisciplinary challenge among participating physicians. Thorough perioperative preparation encompassing monitoring, medical therapy, timing and mode of delivery, and risk consultation is vital in avoiding circumstances that could exacerbate pulmonary arterial hypertension, with physicians readily equipped to promptly detect and manage any untoward event.

## Introduction

Pulmonary arterial hypertension (PAH) is defined as mean pulmonary artery pressure (mPAP) greater than 25 mmHg, with pulmonary capillary wedge pressure, left atrial pressure or left ventricular end-diastolic pressure, less than or equal to 15 mmHg [1]. The upper limit of normal mPAP is proposed as 20 mmHg, and any value above it is considered abnormal. However, this cut-off value does not define a disease per se [2]. As we are aware, peripartum physiological changes may exacerbate any existing heart dysfunction, resulting in significant morbidity and mortality to the mother and fetus [3]. On many occasions, contraception or early termination of pregnancy was recommended [4]. However, patients who present with undiagnosed or misdiagnosed underlying congenital heart disease with severe PAH in their late pregnancy are still received in a Malaysian setting. This case series presents case reports identified from the maternal registry in our local institution, Obstetric Registry of Hospital Canselor Tuanku Muhriz, Universiti Kebangsaan Malaysia on the perioperative management of lower segment caesarean section (LSCS) of five patients with a history of congenital heart disease and severe PAH with a focus on prompt detection and management of any untoward event. The study period was between 1 June 2022 and 31 December 2022. The inclusion criteria were maternity patients presented at the third trimester of pregnancy, mPAP of more than 30 mmHg, and known or unknown status of congenital heart disease. The exclusion criteria were refusal to give consent at the first or second trimester of pregnancy. This study received ethics approval from the Research and Ethics Committee, Universiti Kebangsaan Malaysia Medical Center (JEP-2022-370). Written informed consent was obtained from patients or their next of kin to be included in this case series.

## Case presentation

Case 1

A 34-year-old gravida 2 para 1 (G2P1) woman, who had undergone a surgical repair of the atrial septal defect (ASD) when she was a child but was not on cardiology follow-up, was referred to our centre at 36 weeks of gestation with symptoms of heart failure. Upon admission, she appeared breathless with moderate activity but was comfortable at rest, indicating New York Heart Association (NYHA) Class III. She was immediately transferred to the intensive care unit (ICU) for close observation and treatment. Transthoracic echocardiography showed severe tricuspid regurgitation (TR) with a mPAP of 40 mmHg (Table 1). Oral sildenafil citrate (50 mg daily) and treatments for her heart failure were initiated. A need for urgent LSCS was confirmed in a multidisciplinary discussion among the cardiologist, anaesthesiologist, and obstetrician. During surgery, she received epidural anaesthesia with levobupivacaine 0.5% titrated to a total volume of 10 ml achieving a level of T4 for anaesthesia. Intraoperative monitoring using invasive arterial blood pressure (IABP) and central venous pressure was advocated. During surgery, she was administered noradrenaline infusion and milrinone infusion up to 0.2 mcg/kg/min and 0.5 mcg/kg/min respectively in view of worsening hemodynamic parameters. Unfortunately, she developed a massive postpartum hemorrhage secondary to uterine atony complicated with severe coagulopathy, acute kidney injury, and worsening metabolic acidosis 24 h after surgery. Her condition deteriorated rapidly and succumbed to death at 32 h after LSCS.

**Table 1:** Patients’ demographics parameters. ASD, atrial septal defect; NYHA, New York Heart Association; RA, right atrium; RV, right ventricle; LV, left ventricle; TR, tricuspid regurgitation; LSCS, lower segment caesarean section; WHO, World Health Organization.

	Case 1	Case 2	Case 3	Case 4	Case 5
Status of congenital heart disease	ASD repaired	ASD secundum with bidirectional shunt	ASD with a right-to-left shunt	ASD with a right-to-left shunt	Unknown diagnosis, the defect was repaired
Gestational status on presentation	G2P1 at 36 weeks gestation	G4P1+1 at 32 weeks gestation	G3P2 at 32 weeks gestation	G1P0 at 37 weeks gestation	G1P0 at 32 weeks of gestation
Clinical status	NYHA Class III with failure symptoms	NYHA Class III with failure symptoms	NYHA Class II	NYHA Class I	NYHA Class II
Modified WHO classification of maternal cardiovascular risk	Class IV	Class IV	Class IV	Class IV	Class IV
Echocardiogram findings	Dilated RA with poor RV function but LV function was preserved, severe TR	Dilated RA with poor RV but LV function was preserved, severe TR	Dilated RA, preserved function of RV and LV, severe TR	Dilated RA, preserved function of RV and LV, moderate TR	Dilated RA, preserved function of RV and LV, severe TR
Maximum pressure gradient (estimated based on echocardiogram)	60 mmHg	100 mmHg	90 mmHg	40 mmHg	81 mmHg
Mean pulmonary artery pressure (estimated based on echocardiogram)	60 mmHg	120 mmHg	105 mmHg	50 mmHg	91 mmHg
Anaesthesia technique for LSCS	Epidural anaesthesia	General anaesthesia	Continuous spinal anaesthesia	General anaesthesia	Combined spinal anaesthesia with titrated epidural

Case 2

A 32-year-old G4P1+2 woman was referred to our centre at 32 weeks of gestation for semi-emergency LSCS. She was of NYHA Class III, with dyspnea and tachypnoea on mild exertion. Transthoracic echocardiography showed ASD secundum with bidirectional shunt, severe pulmonary hypertension, and TR (Table 1) thus giving her the diagnosis of Eisenmenger syndrome. She was initiated on oral sildenafil, frusemide, and enoxaparin regularly. Intraoperatively she was monitored using invasive arterial blood pressure and central venous pressure monitoring. LSCS was performed under general anaesthesia with titrated intravenous fentanyl 400 µg, ketamine 50 mg and rocuronium 70 mg during induction. Post endotracheal intubation, the mPAP ranged between 70-80 mmHg measured and estimated using transesophageal echocardiogram. Anaesthesia was maintained with a minimum alveolar concentration (MAC) of 0.8. Phenylephrine bolus doses were titrated, in addition to milrinone and noradrenaline infusion up to 0.3 mcg/kg/min and 0.6 mcg/kg/min respectively, to maintain a mean IABP of 60 mmHg. Uterotonic was given with caution via slow infusion of 100 units of carbetocin over 30 minutes. She was also given a complete reversal with 200 mg of sugammadex after the surgery. Unfortunately, 5 h after LSCS, she developed uterine atony leading to a massive hemorrhage. She was given 100 µg of carbetocin, two units of fresh frozen plasma, and four units of platelet in view of disseminated intravascular coagulation (DIC). Given the worsening mPAP, inhaled nitric oxide was commenced at 8 h after the resuscitation up to 40 ppm. The mean IABP was maintained between 50-60 mmHg with mPAP of 80-90 mmHg. She had a cardiopulmonary arrest 24 h after surgery and resuscitation was futile.

Case 3

A 30-year-old, G3P2 woman had ASD with PAH. She was referred at 32 weeks of gestation and was prescribed oral sildenafil 50 mg daily by the cardiology team. At 36 weeks of gestation, the patient was admitted for LSCS. She was of NYHA Class I with mPAP of 103-105 mmHg (Table 1). LSCS was done under continuous spinal anaesthesia (CSA) with a loading dose of 1.2 mL of 0.5% heavy bupivacaine, 15 µg fentanyl, and two subsequent doses of 0.5 mL 0.5% heavy bupivacaine titrated to achieve anaesthetic level up to T4. A slow infusion of 5 units of oxytocin was commenced upon delivery of the baby. Throughout the surgery, she received noradrenaline and milrinone infusion to improve her cardiorespiratory function. Postoperatively, she was observed in the obstetrics high dependency ward. On day 2, her clinical parameters improved, oral sildenafil was continued, and she was discharged from high high-dependency ward the following day. She was eventually discharged home after days in the obstetric ward.

Case 4

A 33-year-old G1P0 woman was referred to our centre for LSCS at 37 weeks. She had ASD with PAH but had defaulted cardiology follow-up. At the presentation, she was in NYHA Class I with a mPAP of 50 mmHg (Table 1). She was immediately started on oral sildenafil 50 mg daily with LSCS planned 5 days later. Intra-operatively, she received general anaesthesia with intravenous fentanyl 200 µg, ketamine 80 mg, midazolam 2 mg and rocuronium 70 mg with invasive hemodynamic monitoring such as arterial blood pressure and central venous pressure monitoring maintaining between 65-75 mmHg and 8-12 cmH_2_0 respectively. Anaesthesia was maintained with sevoflurane to achieve MAC of 0.8. She was hemodynamically stable intraoperatively without any supportive therapy and was extubated at the end of the surgery. She was transferred to the ICU for close monitoring. A repeat echocardiography showed no new changes, and she was transferred to the ward after 48 h.

Case 5

A 26-year-old G1P0 woman was referred at 32 weeks of gestation for delivery booking. She gave a history of heart surgery at 1 year of age with her cardiology assessment showing a severe PAH (Table 1). She was unable to recall the diagnosis of her congenital heart disease. She was of NYHA Class I with mPAP 91 mmHg. She was prescribed oral sildenafil 50 mg daily and was planned for LSCS at 36 weeks of gestation. She received epidural anaesthesia with 15 μg fentanyl, 1.0 mL of 0.5% heavy bupivacaine, and a total of 3 mL of 0.5% levobupivacaine given through epidural which was titrated to effect. Her respiratory and hemodynamic parameters were stable intraoperatively without any supportive therapy. She was monitored and observed in the high-dependency unit after the surgery and was discharged to the ward after 2 days.

## Discussion

Based on the latest guideline of the American Heart Association (AHA) pulmonary hypertension includes patients with mPAP more than 20 mmHg [5]. All five patients presented with gestational age ranging between 32-37 weeks, with their mPAP ranging between 50-110 mmHg, when the decision for LSCS was made. The most critical of the five patients had a bidirectional ASD shunt with a mPAP of 110 mmHg (Case 2). She was in heart failure at presentation and required additional vasopressors intraoperatively following anaesthesia induction. Postoperative complications lead to massive hemorrhage and coagulopathy further complicating her condition. She also received nitric oxide inhalation but her mPAP remained high and resuscitation was futile. In contrast, the other patient (Case 1) who succumbed to death had her ASD repaired, a lower mPAP of 60 mmHg, with preserved left ventricle function. Unfortunately, she developed coagulopathy and acute kidney injury with severe metabolic acidosis post-LSCS. Two of our patients had mPAP above 100 mmHg but only one ended with mortality. Despite different modes of anaesthesia, one died. Regardless of the initial mPAP, careful delivery of the mode of anaesthesia, and diligent perioperative monitoring and resuscitation in all patients, fatality was inevitable in two of the five patients. It was reported that despite therapy, the prognosis was poor with the incidence of maternal mortality ranging between 30% and 50% [4]. The fatalities in this report had severe postpartum haemorrhage with existing poor right ventricle (RV) function and PAH which may have complicated the resuscitation. There might be a few steps that we could take to reduce the risk of postpartum haemorrhage such as promoting regional anaesthesia as compared to general anaesthesia, use of uterotonics is also advocated but with caution, meticulous surgical technique to ensure good hemostasis and lastly the use of point of care testing to check patient coagulation status using thromboelastogram. 

Pregnancy-related cardiovascular changes which include an increase in blood volume and cardiac output, reduction in systemic vascular resistance, physiological anaemia and hypercoagulability, may camouflage the symptoms and signs of PAH and delay the diagnosis and therapy worsening the prognosis [4]. Venous return is progressively impeded by the enlarging uterus impairing the right ventricular filling and further compromising the right ventricular output. Advanced functional class, poor exercise tolerance, high right atrial (RA) pressure, significant RV dysfunction and failure are predictors of poor prognosis [1]. Two of our patients who died postoperatively had presented with advanced NYHA functional class, poor RV function, cor pulmonale, and post-partum haemorrhage. The surviving patients had preserved RV function despite high pulmonary artery pressures, with no other poor prognostic factors and post-LSCS complications.

Patients with PAH should be managed by a multidisciplinary team comprising an obstetrician, cardiologist, anaesthesiologist, intensivist, and neonatologist [4]. Regular follow-up is advocated, with timely echocardiography and fetal surveillance. All the patients in this report were not on regular antenatal follow-up and were seen by the cardiology team for the first time upon referral. Right heart catheterization remains a gold standard assessment of hemodynamic parameters in pulmonary hypertension [3]. Perioperative use of minimally invasive cardiac output monitoring has been advocated such as transesophageal Doppler ultrasound. However, this device will only derive estimated values of PAP in these patients [4].

Elective admission for treatment optimization up to delivery may be necessary [6]. Patients with well-controlled PAH on therapy, and low pulmonary vascular resistance responsive to calcium channel blockers, may have their risks lowered [7]. Sildenafil acts on the nitric oxide signaling pathway and selectively inhibits phosphodiesterase-5 in the lung tissue potentially mediating relaxation and inhibiting vascular smooth muscle cell growth. It reduces symptoms, improves effort tolerance and functional class, reduces PAP, and controls refractory heart failure following 2 weeks of treatment [8,9]. Prostacyclin analogues, which include intravenous epoprostenol, subcutaneous treprostinil, and aerosolized iloprost, which were unavailable in the case described here, have demonstrated benefits in treating pulmonary hypertension [3]. The use of diuretics and low molecular weight heparin in reducing fluid overload for cor pulmonale and as an anticoagulant, respectively are well recommended [9]. Patients in our report presented late in their third trimester and sildenafil was initiated only for a few days except for two patients (Cases 3 and 5) who were prescribed sildenafil approximately 4 weeks prior to their LSCS.

The recommendations regarding the optimum mode of delivery, anaesthesia and analgesia, the extent of heart monitoring, and peripartum management remain controversial. Regardless of the delivery mode, it is imperative that PAH needs to be well-controlled. Prolonged labour with a consequent increase in cardiac output and venous return can be detrimental [10]. Nitrous oxide as labour analgesia should be avoided as it may constrict the pulmonary vasculature [10]. Planned CS at a gestational age between weeks 32 and 36 is a compromise between maternal health and sufficient fetal maturation and reduces the risk of spontaneous labour at unsocial hours [10]. The patients described here were scheduled for LSCS at 32-36 weeks of gestation.

Both regional and general anaesthesia require caution to minimize vasodilatation and exacerbation of the compromised cardiovascular status. Epidural or combined spinal epidural (CSE) anaesthesia is well tolerated [2,3]. Low-dose sequential CSE or CSA permits gradual titration of a local anaesthetic to effect while obviating undesired precipitous reductions in blood pressure. General anaesthesia allows some control over anaesthetic titration; however, the crucial moments of intubation, extubation, intraoperative awareness, and pain, as well as mechanical ventilation, should be optimally managed to minimize the adverse effects of sympathetic stimulation, hypoxia, as well as hypercarbia and acidosis, which risks elevated PAP. In our patients with septal defects, shunt reversal and consequent hypoxia could ensue from the increased PAP. Two of our cases received general anaesthesia while the other received central neural blockade. There is no textbook answer for us to decide which technique is better than the other, hence it has to go on a case-by-case basis and as long as we are able to justify why one technique is preferred to the other, we can never go wrong. Oxytocin remains the first-line uterotonic post-delivery, but caution needs to be exercised as hypotension and reflex tachycardia potentially increase PAP [11]. Post-partum monitoring is equally important as most deaths occur during this period, and monitoring should continue from days to weeks following delivery [3,7]. The highest risk of mortality lies in the 4 weeks postdelivery with cor pulmonale accounting for a majority of the deaths [4,7].

## Conclusions

In conclusion, the management of severe pulmonary arterial hypertension (PAH) in pregnant patients remains a multidisciplinary challenge among participating physicians. Thorough perioperative preparation encompassing monitoring, medical therapy, timing and mode of delivery as well as risk consultation is vital. Perioperative management is focused on avoiding all circumstances that could exacerbate PAH, with physicians readily equipped to promptly detect and manage any untoward event.
